# Precision Medicine and Global Health: The Good, the Bad, and the Ugly

**DOI:** 10.3389/fmed.2018.00067

**Published:** 2018-03-14

**Authors:** Alexios-Fotios A. Mentis, Kleoniki Pantelidi, Efthimios Dardiotis, Georgios M. Hadjigeorgiou, Efthimia Petinaki

**Affiliations:** ^1^The Johns Hopkins University, Advanced Academic Programs (AAP), Baltimore, MD, United States; ^2^Department of Microbiology, University Hospital of Larissa, University of Thessaly, Larissa, Greece; ^3^Department of Neurology, University Hospital of Larissa, University of Thessaly, Larissa, Greece

**Keywords:** precision medicine, genomics, sequencing, big data, global health, health policy, health equity, social determinants of health

Precision medicine holds significant promise for finding new ways to diagnose, prevent, and treat disease. In October 2017, Bill Gates and Francis Collins, thought-leaders in global health and genomics, respectively, jointly discussed shared perspectives in these arenas (1). A lot of the promise of precision medicine has yet to be realized (2), so, it is important that the global health community, itself facing challenges, enters this new era with its eyes wide open.

There are concerns that a focus on precision medicine may detract attention from the need to tackle the social and environmental determinants of health (3). Lying as they do at the heart of health inequity, these determinants must be tackled as a leading global health priority [such as the United Nation’s Sustainable Development Goals (SDGs) of “no poverty” and “zero hunger”]. At first glance, the SDG goal to “leave no one behind” seems aligned with precision medicine in terms of intent to provide the best treatment to all patients. Even if, as expected, the cost of high-throughput DNA sequencing falls to such a level to allow population-wide personal genomics, it is likely that this technology will remain a first-world pursuit rather than becoming available to all. This is because developed countries can afford advanced treatments and because research is directed into diseases that most affect these populations. In that sense, the knowledge garnered from global genetic analyses could further widen global health disparities.

Perhaps of greater concern, genetic testing may detract from low-tech but proven public health measures in resource-limited settings (4). Focus must remain on implementing policy that is known to be effective rather than conducting research for research’s sake (5). Maintaining this focus is the first step to achieving population-wide health prevention; for example, tobacco taxation is likely to be of greater benefit than pharmacogenomics for tobacco cessation. Social policy should not be regarded as less scientifically challenging than advanced technologies. Adopting a public health-aware approach does not fundamentally contradict or negate the need to fund research into diseases with well-defined genetic backgrounds (e.g., cancer or genetic syndromes) (6, 7) to inform future precision therapy, but basic public health principles must not be forgotten.

It has recently been argued that computers can process complex diagnostic and treatment decisions more efficiently than the human brain (8), and discounting technology’s value would be folly; indeed, artificial intelligence-inspired medical practice could usher in a new era of global health. However, precision medicine runs the risk of placing too much emphasis on algorithms and not taking the patient’s complex background and needs, such as culture, values, preferences, and beliefs, into consideration. These less quantifiable factors, alongside the fear of “losing the patient from the picture,” are a particular concern for all health systems, not least in resource-poor settings (4). Hence, computers are perhaps better regarded as “*thinking partners”* (8) to avert removing the *personal* element of *personalized* treatment.

Finally, genomic information usually informs us about the predisposition to, but not the complete risk of, developing a disease or responding to therapy (2). The entirety of biological information from genome to proteins to organism, their highly complex interactions, and the biological effects of environmental factors, have yet to be deciphered and may explain the genotype–phenotype gap. Although conducting extensive biological analyses (i.e., -omics analysis of proteins and metabolites) may be deemed a research priority, it is questionable whether that level of precision will be clinically meaningful, cost-effective, and financially sustainable given the competing priorities in developing countries.

Therefore, we emphasize the need to remain focused on leading health-care priorities and bold interventions. Precision medicine in the “big data” era presents attractive opportunities to improve health care worldwide, but only if framed within the wider societal and global context. Although it is tempting to do so, we should not become fanatical about precision medicine or see it as a panacea, and it needs to be balanced with population-centered approaches.

## Author Contributions

AAM and KP conceived the study and wrote the first draft. ED, GMH, and EP supervised the study and revised the draft. All authors have read and approved the final draft.

## Disclaimer

AAM is supported by an educational scholarship from the Onassis Public Benefit Foundation, who played no role in the design of the study, or writing of this Opinion.

## Conflict of Interest Statement

The authors declare that the research was conducted in the absence of any commercial or financial relationships that could be construed as a potential conflict of interest.
