# Corrigendum: Precision Medicine and Global Health: The Good, the Bad, and the Ugly

**DOI:** 10.3389/fmed.2018.00095

**Published:** 2018-04-11

**Authors:** Alexios-Fotios A. Mentis, Kleoniki Pantelidi, Efthimios Dardiotis, Georgios M. Hadjigeorgiou, Efthimia Petinaki

**Affiliations:** ^1^The Johns Hopkins University, Advanced Academic Programs (AAP), Baltimore, MD, United States; ^2^Department of Microbiology, University Hospital of Larissa, University of Thessaly, Larissa, Greece; ^3^Department of Neurology, University Hospital of Larissa, University of Thessaly, Larissa, Greece

**Keywords:** precision medicine, genomics, sequencing, big data, global health, health policy, health equity, social determinants of health

In the original Opinion article, there was an error in the affiliation address of the first and corresponding author.

In the affiliations section, the phrase “American Academy of Pediatrics (AAP)” was mistakenly placed, and it has now been replaced with the correct phrase “Advanced Academic Programs (AAP).” The authors sincerely apologize for the error.

This error does not change the scientific conclusions of the Opinion Article in any way.

The original article has been updated.

## Conflict of Interest Statement

The authors declare that the research was conducted in the absence of any commercial or financial relationships that could be construed as a potential conflict of interest.

